# A comparative study of ultrasound-guided versus landmark-based femoral with sciatic nerve blocks in lower limb surgeries

**DOI:** 10.6026/973206300223457

**Published:** 2026-06-30

**Authors:** Ajita Tiwari, Irfan Ahmad Siddiqui, Sushil Chand Verma

**Affiliations:** 1 Department of Anaesthesia, Birsa Munda Government Medical College, Shahdol, Madhya Pradesh, India; 2 Department of Anaesthesia, Government Medical College, Satna, Madhya Pradesh, India

**Keywords:** Peripheral nerve block, ultrasound guidance, landmark technique, lower limb surgery, regional anesthesia

## Abstract

Peripheral nerve blocks are widely used in lower limb surgeries to provide effective anesthesia and post-operative analgesia. Therefore, it is of interest to compare ultrasound-guided and landmark-based femoral with sciatic nerve block techniques in terms of success rate, onset time, duration of analgesia and complications. A prospective comparative study was conducted on 100 patients undergoing elective lower limb surgeries, divided equally into ultrasound-guided and landmark-based groups. Ultrasound-guided blocks demonstrated significantly faster onset, higher success rates, longer duration of analgesia and fewer complications compared to the landmark-based technique (p < 0.05). Thus, data shows that ultrasound guidance improves the safety, accuracy and overall efficacy of peripheral nerve blocks in clinical practice.

## Background:

Peripheral nerve blocks are an essential component of modern regional anesthesia, particularly femoral with sciatic nerve block in lower limb surgeries, due to their ability to provide targeted anesthesia with minimal systemic effects [1]. Traditionally, landmark-based techniques have been used; however, these rely heavily on anatomical knowledge and may be associated with variable success rates and complications [2]. The use of ultrasound guidance has significantly enhanced regional anesthesia by enabling real-time visualization of neural structures, adjacent tissues and needle positioning [3]. Recent studies have demonstrated improved block accuracy, reduced onset time and decreased complications with ultrasound-guided techniques compared to conventional methods [4]. Despite these advantages, the adoption of ultrasound guidance in resource-limited settings remains inconsistent [5, 6]. Therefore, it is interest to compare ultrasound-guided and landmark-based femoral with sciatic nerve blocks in lower limb surgeries to assess their clinical effectiveness and safety.

## Methods:

A prospective, randomized comparative study was conducted in the Department of Anesthesiology at a tertiary care center. A total of 100 patients undergoing elective lower limb surgeries were included in the study.

## Inclusion criteria:

[1] Patients aged 18-65 years

[2] ASA physical status I and II

[3] Scheduled for elective lower limb surgery

[4] Patients providing informed consent

## Exclusion criteria:

[1] Coagulopathy or bleeding disorders

[2] Infection at injection site

[3] Allergy to local anesthetics

[4] Neurological disorders affecting lower limbs

[5] Refusal to participate

## Grouping:

[1] Group A (n=50): Ultrasound-guided femoral with sciatic nerve block

[2] Group B (n=50): Landmark-based femoral with sciatic nerve block

## Parameters assessed:

[1] Block onset time

[2] Block success rate

[3] Duration of analgesia

[4] Complications (vascular puncture, nerve injury, local anesthetic toxicity)

## Statistical analysis:

Data were analyzed using SPSS version 26. Continuous variables were expressed as mean ± standard deviation and compared using Student's t-test. Categorical variables were analyzed using Chi-square test. A p-value <0.05 was considered statistically significant.

## Results:

A total of 100 patients were included in the study and divided equally into Group A and Group B (50 patients each). The demographic variables such as age, gender and ASA status were comparable between the two groups. No statistically significant difference was observed (p > 0.05). This indicates that both groups were similar at baseline (Table 1). Group A showed a significantly faster onset of block compared to Group B (p <0.001). The success rate was significantly higher in Group A, indicating superior efficacy of the ultrasound-guided technique (Table 2). The duration of analgesia was significantly longer in Group A compared to Group B (p <0.001). This suggests improved post-operative pain control with the ultrasound- guided approach (Table 3). The incidence of complications was lower in Group A compared to Group B. A statistically significant difference was observed for vascular puncture (p < 0.05), while other complications were not statistically significant (Table 4). Figure 1 (see PDF) demonstrates a significantly faster onset time in Group A compared to Group B.

## Discussion:

Recent developments in regional anesthesia have highlighted the importance of ultrasound guidance in enhancing the precision and reliability of nerve block techniques [7]. In the present study, ultrasound-guided femoral with sciatic nerve blocks demonstrated a significantly faster onset time compared to landmark-based techniques, which is consistent with findings from recent studies [8]. The higher success rate observed in the ultrasound group can be attributed to direct visualization of neural structures and accurate local anesthetic deposition [9]. Additionally, the reduced incidence of complications such as vascular puncture further supports the safety advantage of ultrasound guidance [10]. Recent studies have also highlighted improved patient outcomes and reduced procedural variability with ultrasound use [11]. Despite this, the requirement for equipment and training may limit its widespread use in certain healthcare settings [12, 13]. The findings of this study have important clinical implications, suggesting that the use of ultrasound guidance can enhance procedural efficiency, improve patient safety and optimize perioperative outcomes in lower limb surgeries. However, this study has certain limitations, including a relatively small sample size and single-center design, which may limit the generalizability of the findings.

## Conclusion:

Ultrasound-guided femoral with sciatic nerve blocks are superior to landmark-based techniques in lower limb surgeries, offering faster onset, higher success rates, prolonged analgesia and fewer complications. Incorporating ultrasound guidance into routine anesthetic practice can significantly improve patient outcomes and procedural safety. Thus, efforts should be made to enhance accessibility and training to facilitate its broader implementation.

## Figures and Tables

**Table 1:** Demographic characteristics of patients in Group A and Group B

**Parameter**	**Group A**	**Group B**	**p-value**
Age (years)	42.5± 10.2	43.1± 9.8	0.78
Gender (M/F)	28/22	30/20	0.67
ASA I/II	32/18	30/20	0.64

**Table 2:** Comparison of block characteristics between Group A and Group B

**Parameter**	**Group A**	**Group B**	**p-value**
Onset Time (min)	8.2 ± 2.1	15.4 ±3.6	<0.001
Success Rate (%)	96%	82%	0.02

**Table 3:** Comparison of duration of analgesia between Group A and Group B

**Parameter**	**Group A**	**Group B**	**p-value**
Duration (hours)	10.5 ± 2.3	7.8 ± 2.0	<0.001

**Table 4:** Comparison of complications between Group A and Group B

**Complication**	**Group A**	**Group B**	**p-value**
Vascular puncture	2%	10%	0.04
Nerve injury	0%	4%	0.15
LA toxicity	0%	2%	0.31
